# The expression and prognostic impact of CXC-chemokines in stage II and III colorectal cancer epithelial and stromal tissue

**DOI:** 10.1038/sj.bjc.6606055

**Published:** 2011-02-01

**Authors:** O Oladipo, S Conlon, A O'Grady, C Purcell, C Wilson, P J Maxwell, P G Johnston, M Stevenson, E W Kay, R H Wilson, D J J Waugh

**Affiliations:** 1 Centre for Cancer Research and Cell Biology, Queen's University Belfast, 97 Lisburn Road, Belfast BT9 7BL, Northern Ireland; 2 Department of Histopathology, Royal College of Surgeons in Ireland, Beaumont Hospital, Dublin, Ireland; 3 Centre for Public Health, Queen's University Belfast, Belfast BT9 7BL, Northern Ireland

**Keywords:** CXCL1, CXCL8, CXCR1, CXCR2, colorectal cancer

## Abstract

**Background:**

The CXC-chemokine expression is linked with colorectal cancer (CRC) progression but their significance in resected CRC is unclear. We explored the prognostic impact of such expression in stage II and III CRC.

**Methods:**

Tissue microarrays were constructed from stage II and III CRC biopsies (*n*=254), and the expression of CXCL1 and CXCL8, and their receptors CXCR1 and CXCR2, in malignant and adjacent normal tissue was graded by immunohistochemistry and was correlated with prognostic factors.

**Results:**

Expression of CXCL1, CXCR1 and CXCR2 was elevated in tumour epithelium relative to normal adjacent tissue (*P*<0.001). CXCL8 expression was detectable in the peritumoural inflammatory infiltrate. There was no overall association between CXCL1, CXCR1 or CXCR2 expression and prognostic endpoints; however, univariate subgroup survival analysis demonstrated an inverse association between CXCL1 and recurrence-free survival (RFS) in stage III patients (*P*=0.041). The CXCL8 positivity in the tumour infiltrate, however, correlated with earlier disease stage (*P*<0.001) and improved relapse-free survival across the cohort (*P*<0.001). Disease stage (*P*<0.001) and tumour infiltrate CXCL8 positivity (*P*=0.007) were associated with enhanced RFS in multivariate Cox regression analysis.

**Conclusion:**

Autocrine CXC-chemokine signalling may have adverse prognostic effects in early CRC. Conversely, CXCL8 positivity within the immune infiltrate may have good prognostic significance.

Colorectal cancer (CRC) remains a major cause of morbidity and mortality in the Western world, accounting for more than one in eight newly diagnosed cancers in Europe and over half a million deaths worldwide annually (Parkin *et al*, 2005; Ferlay *et al*, 2007). Despite this, only half of the patients who undergo potentially curative surgery survive for 5 years (McArdle and Hole, 2002), and the challenge of how best to predict prognosis and, thereby optimise therapy remains. At present, established clinico-pathological criteria are used to estimate risks of recurrence in stage II and III disease, and this is routinely used in the selection of patients for adjuvant systemic therapy following surgical resection. The clinical outcome of patients who receive such adjuvant treatment can, however, vary widely, when additional molecular factors are taken into consideration (Allegra *et al*, 2003). Identification of novel prognostic markers is, therefore, vital in improving the prognosis of this disease.

Chemokines are a large family of chemotactic signalling molecules that are increasingly attracting attention in the study of tumourigenic mechanisms within malignant cells and the tumour microenvironment. They are classified into four broad groups as follows: C, CC, CXC and CX_3_C, on the basis of the position of their cysteine residues (Payne and Cornelius, 2002). The CXC-chemokines are subdivided further into ELR+ and ELR−, depending on the amino-acid sequence before the first cysteine residue, and this confers angiogenic or angiostatic potential on these molecules (Strieter *et al*, 1995). The best characterized and prototypic CXC-chemokine is CXCL8, previously termed interleukin-8 (Rollins, 1997; Beck *et al*, 1999) and its biological effects are mediated through two G-protein-coupled receptors designated CXCR1 and CXCR2. A series of structurally related CXC-chemokines, including CXCL1, also bind selectively to the CXCR2 receptor (Ahuja and Murphy, 1996). Both CXCL1 and CXCL8 are pro-inflammatory mediators, functioning as chemotactic factors for neutrophils (Schroder *et al*, 1990a).

Multiple clinical studies implicate CXC-chemokines in the development and progression of CRC. For example, elevated tumour CXCL8 levels have been associated with increased tumour size, depth of infiltration, disease stage and liver metastasis, as well as a shorter overall survival time in CRC (Terada *et al*, 2005). Furthermore, the circulating level of CXCL8 in patients’ serum is higher in more advanced disease stage and in the presence of bowel wall invasion, liver and/or lung metastasis (Ueda *et al*, 1994; Kaminska *et al*, 2005). The CXCL1 and its receptor CXCR2 are also widely reported to be elevated in CRC, the presence of which may facilitate CRC tumour progression (Erreni *et al*, 2009). However, none of these studies have specifically studied the expression profile of these chemokines and their receptors in stage II and III CRC patients who have had potentially curative resection. Thus, their potential as prognostic markers in this disease is unclear.

Chemokine signalling not only modulates the function of cancerous epithelial cells but also serves as an intermediary in the communication network between tumour cells and the surrounding stroma. CXC-chemokines have potent effects in recruiting immune cells to inflammatory sites and CXCL1 and CXCL8 are both associated with neutrophil recruitment and activation (Moser *et al*, 1990; Schroder *et al*, 1990a). Interestingly, assessment of the peritumoural inflammatory infiltrate has been shown to provide prognostic information in CRC (Roxburgh *et al*, 2009), raising interest in the study of their clinical implications in this and other solid tumours. Characterisation of CXC-chemokine expression in these tumour-infiltrating cells is however limited, whereas the clinical relevance of such expression remains uncertain.

Our study presents an immunohistochemical profile of the expression of CXC chemokines and their receptors in 254 stage II and III CRC tissues. The expression of CXCL1 and CXCL8 and their receptors CXCR1 and CXCR2 were evaluated within diseased epithelium and compared with adjacent normal epithelium in patient-matched samples. Additionally, CXCL8 expression was characterized throughout the immune infiltrate of the tumours. We report an increased expression of CXCL1, CXCR1 and CXCR2 in tumour, compared with the surrounding normal epithelium, providing evidence for enhanced autocrine CXC chemokine signalling in these cancer cells. Furthermore, we determine a range of CXCL8-positivity within the immune cell-infiltrate, with important clinical implications. Accordingly, the significance of epithelial tumour and tumour immune cell infiltrate expression of these markers is presented.

## Materials and methods

### Patients and specimens

Archived formalin-fixed and paraffin-embedded CRC tissue specimens of 254 patients with stage II and stage III CRC were retrieved for the study. The specimens were originally obtained during a randomised controlled phase III clinical trial comparing 16 weeks of De Gramont schedule FU/FA to observation alone in patients with stage II and III CRC (24). Patients were recruited from hospitals in Northern Ireland for this study between March 1994 and February 1997, in compliance with the tenets of the Declaration of Helsinki. The trial participants gave informed consent for the use of their tissue for further molecular biomarker studies, such as this, and ethical approval for this study was obtained from the local Ethics Committee.

### Tissue microarray construction

The study was performed using archival, formalin-fixed paraffin-embedded colon tumour samples. A total of 254 primary colon carcinoma cases were selected.

All formalin-fixed paraffin-embedded blocks were sectioned and stained with H&E and graded by a pathologist (EWK) to confirm pathological stage and grade of the tumours, and the relevant tumour areas were marked and used as the donor cores for TMA construction. The construction was performed using the Beecher Instruments Tissue Microarrayer (Beecher Instruments, Silver Spring, MD, USA) (Kononen *et al*, 1998). Cores of 0.6 mm thickness were sampled in quadruplicate for each case.

### Immunohistochemistry

Sections of 4 *μ*m thickness were cut from all TMAs for the purpose of immunohistochemistry.. Sections were immunostained with CXCL1 (R&D Systems, Abingdon, UK, cat# MAB275), CXCR1 (Biosource, Invitrogen, Carlsbad, CA, USA, cat# AHR1522X), CXCR2 (Biosource, cat# AHR1532X) and CXCL8 (Affinity Bioreagents, Golden, CO, USA, cat# PA1-32883) on an automated platform (Bond system – Leica Microsystems, Bannockburn, IL, USA). Briefly, cut sections were subjected to on-board dewaxing and the following conditions: CXCL1 and CXCR2 – antigen retrieval in tri-sodium citrate buffer (Bond Epitope Retrieval 1 solution) for 10 min and 1 : 500 antibody dilution; CXCR1 – antigen retrieval in EDTA buffer (Bond Epitope Retrieval 2 solution) for 20 min and 1 : 4000 antibody dilution; CXCL8 – no antigen retrieval and 1 : 500 antibody dilution. Detection of the antibody–antigen complex was achieved using a polymer-based kit (Bond Refine) with DAB as the chromogen. All sections were counterstained with haematoxylin. Negative controls were included for all sections by omitting the primary antibody and positive controls used included samples of tonsil and colonic adenocarcinoma.

### Immunohistochemical assessment

Two reviewers who were blinded to the clinico-pathological details and clinical outcome of the cohort performed the immunohistochemical evaluation of the staining independently. Cores with at least 50% of the tissue preserved after sectioning were included in the study. The TMAs were stained for expression of the CXC-chemokine receptors CXCR1 and CXCR2, and the ligands CXCL1 and CXCL8. Cytoplasmic epithelial staining of the tissue cores for CXCR1, CXCR2 and CXCL1 was detected and the degree of expression was recorded. This was determined by a combined score comprising the percentage of cells with staining (none=0; <10%=1; 10–50%=2; 51–80%=3; >80%=4) and the intensity of the staining (none=0; weak=1; moderate=2; strong=3). The product of both values was described as the immunoreactivity score (IRS), and used for the final analysis (Remmele and Stegner, 1987); this method has been used in similar studies evaluating chemokine immunohistochemistry expression (Eck *et al*, 2003; Jiang *et al*, 2006). Colorectal cell epithelial CXCL8 staining was minimal and, therefore, formal evaluation was not performed.

Immunostaining of the surrounding infiltrating inflammatory cells was also observed, in relation to both malignant and normal tissue. Although the epithelial tissue had shown negligible staining for CXCL8, this infiltrate demonstrated clear differential staining, particularly within the tumour cores. Consequently we evaluated CXCL8 expression within these inflammatory cells, in order to explore the clinical implications of the differences in staining. Because of the limited size of the tissue cores and as the immunostaining in the inflammatory cells appeared homogenous, the scoring system used for the colorectal cells was deemed inappropriate for the infiltrate. Therefore, each core was denoted as either negative or positive for inflammatory cell CXCL8 expression. Only two of the cases evaluated had no visible inflammatory cells within their available cores; these were, therefore, regarded as negative. Cases with at least two positive staining cores were considered positive in the final analysis. There was strong correlation between the two scorers, and in discrepant cases, a consensus was reached after a joint review.

There was some sample loss through damage to the cores during TMA construction and sectioning, a well-recognised limitation of the procedure. Tissue damage rates ranging from 15 to 33% have been reported (Schraml *et al*, 1999; Mucci *et al*, 2000; Richter *et al*, 2000), and this study was able to recover 72% of cases for evaluation of the various markers, a proportion thus comparable to the literature.

### Statistics

Patient data had been collected via a centralised trial co-ordinating office during the period of the clinical study and stored electronically. Statistical analysis was performed via the SPSS 17 and SPSS 18 software packages (SPSS, Chicago, IL, USA). Differences between the mean IRS scores of the malignant and normal colorectal epithelium samples were compared using the paired Student's *t*-test. Correlation of CXC-chemokine and receptor expression was performed using Spearman rank correlation test. Association between the IRS scores and clinico-pathological variables was assessed using *χ*^2^-test and Fisher's exact test as appropriate. Survival rates were compared using the Kaplan–Meier curves, and univariate survival analysis performed using the log-rank test. Multivariate survival analysis was performed through Cox regression analysis. A *P*-value of <0.05 was considered significant in all analyses performed.

## Results

### Clinical-pathological features

The median age of the patients at the time of surgery was 64 years (range 35–80 years). Males (*n*=149) and females (*n*=105) were included in the study cohort and the median duration of follow up was 68 months (range 11–105 months). In total, 71 patients had rectal carcinoma, whereas 183 had colonic carcinoma. There was a predominance of patients with stage II disease among the patients (163 patients with stage II and 91 patients with stage III disease). Table 1 shows the clinico-pathological demographics of the patient cohort, along with the incidence of recurrence and survival status in the group.

### Comparison of CXCR1, CXCR2, CXCL1 and CXCL8 expression within normal and malignant colorectal tissue

**Figure 1:**
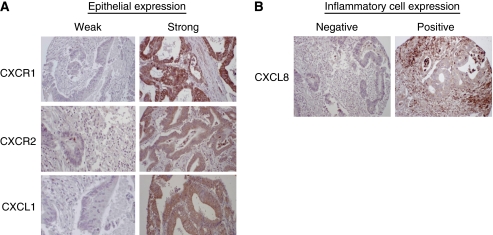
Immunohistochemical characterisation of CXC-chemokine and its receptor expression in colorectal tissue. (**A**) Representative high-powered images (magnification × 200) illustrating weak (left panel) and strong (right panel) immunoreactivity to antibodies used to characterize the expression of CXCR1, CXCR2 and CXCL1 in colorectal biopsy tissue. (**B**) Representative low-powered images showing differential expression of CXCL8 within the inflammatory cells surrounding the colorectal epithelial tissue.

**Figure 2:**
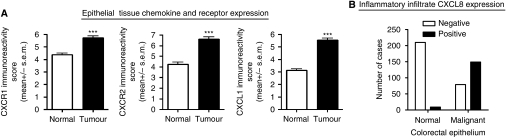
Quantitative comparison of CXC-chemokine and its receptor expression in colorectal tissue. (**A**) Bar graphs presenting the relative immunoreactivity score calculated for CXCR1, CXCR2 and CXCL1 in normal and malignant colorectal epithelial cells. The mean expression in tumor and normal tissues was compared using Student's *t*-test. Malignant tissue showed significantly higher levels of expression of all three markers in comparison to the surrounding normal tissue; ^***^P<0.001. (**B**) Bar graph illustrating the number of positive cases in which CXCL8 immunoreactivity was detected in the inflammatory cells surrounding the normal and malignant epithelial cells in the colorectal tissue. The majority of the cores with normal epithelium are absent for CXCL8 expression within the inflammatory infiltrate. In contrast, a greater number of cores with malignant epithelium show inflammatory cell CXCL8 positivity.

The tissue specimens were evaluated for immunohistochemical expression of the CXC-chemokines CXCL1 and CXCL8 and their receptors CXCR1 and CXCR2 within the neoplastic and surrounding normal colorectal epithelium. Differences in the degree of expression of these markers within the cytoplasm of colorectal epithelial cells are illustrated in Figure 1A. The mean expression of CXCR1, CXCR2 and CXCL1 for each case was classified as absent or weak (IRS 0–3), moderate (IRS between three and nine) and strong (IRS nine and above) as has been previously described (Jiang *et al*, 2006). All the three markers showed predominantly weak to moderate expression within normal colorectal epithelium; conversely moderate to strong expression of each marker was exhibited by the colorectal tumour epithelium (Supplementary Table S1). Comparison of the mean IRS scores for CXCR1, CXCR2 and CXCL1 in malignant and non-malignant tissue confirmed higher expression of each chemokine marker in the tumour epithelium relative to adjacent normal colorectal tissue (Figure 2A). Additionally, correlation of the chemokine/receptor expression within the malignant epithelium demonstrated a modest, but statistically significant correlation between the expression of CXCL1 and its receptor CXCR2 in the cohort of patients (*r*=0.263; *P*<0.001; Supplementary Table S2).

There was no meaningful immunostaining for CXCL8 within the malignant or normal colorectal epithelium and this was, therefore, not included in the epithelial tissue analysis; however, we detected a significant and differential staining of CXCL8 within the infiltrating inflammatory cells in the tissue sections (Figure 1B). The majority of the tumour cores (65.4%) expressed positive CXCL8 immunoreactivity within the tumour-associated inflammatory infiltrate. Conversely, 95.9% of the normal colorectal tissue cores had no detectable CXCL8 expression within their infiltrating inflammatory cells (Figure 2B).

### Association between CXCR1, CXCR2, CXCL1 and CXCL8 expression and clinico-pathological factors

The association between CXCR1, CXCR2 and CXCL1 and CXCL8 expression in the tumour tissue and the various clinico-pathological features of the study cohort is provided in Table 2. We observed a significantly higher level of CXCR1 epithelial expression in rectal cancer tissue compared with colon, with 18.6% of rectal tumour cases showing strong expression compared with 7.3% of colon cancer cases (*P*=0.033). There was no significant difference in epithelial expression of the other markers between tumour sites. Although there was no statistically significant correlation between disease stage and neoplastic epithelial expression of the chemokine markers, a higher proportion of stage II patients had CXCL8-positive inflammatory cell infiltrate within tumour cores, compared with stage III patients (*P*=0.031).

No association was found between tumour epithelial immunostaining for CXCR1, CXCR2 and CXCL1 or the tumour infiltrate expression of CXCL8 and age at diagnosis, disease stage, pathological grade or patient gender. Statistical analysis was performed using *χ*^2^-test or Fisher's exact test, as appropriate.

### Association with clinical outcome

**Figure 3:**
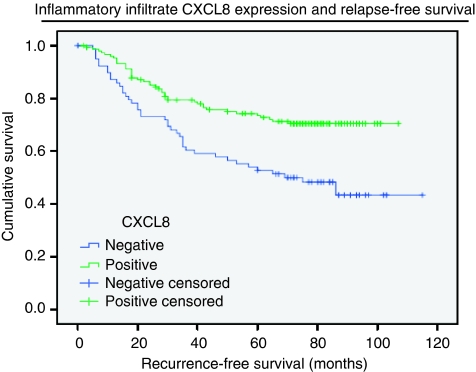
Relapse-free survival of stage II and III CRC patients, based on CXCL8 expression within the tumour inflammatory infiltrate. Kaplan–Meier curve representing the recurrence-free survival of stage II and stage III colorectal cancer patients. Samples were stratified on the basis of either positive or negative CXCL8 immunoreactivity within the infiltrating immune cells surrounding the tumor epithelium. Patients with a CXCL8 positive infiltrate were shown to have an increased relapse-free survival with the median interval not being reached. In contrast, patients with no CXCL8 immunoreactivity had a median survival of 69 months (*P*<0.01; log-ranks test).

The primary objective of the clinical trial was to investigate the impact of 16 weeks of adjuvant 5FU chemotherapy on the clinical outcome of the patient cohort. In this analysis, there was no significant difference between the treatment and observation arms with respect to relapse-free and overall survival (Supplementary Figure S1). The median RFS and overall survival in each treatment arm were not reached at the time of analysis, and there was no statistically significant difference between the Kaplan–Meier curves (*P*=0.23 and *P*=0.38, respectively, log-rank test). Additionally, in the current study the prognostic significance of CXC-chemokine ligand and receptor expression within CRC tissue was also determined, using univariate and multivariate survival analyses. Univariate analysis of the entire cohort (Table 3) showed that disease stage (*P*<0.001), and interestingly, expression of CXCL8 within the tumour inflammatory infiltrate (*P*<0.001) was the only significant factors impacting on recurrence-free survival (RFS). The Kaplan–Meier survival curve (Figure 3) demonstrated that patients with positive CXCL8 staining in this tumour infiltrate had a significantly improved RFS (median survival not reached) compared with patients with negative staining (median survival 69 months; 95% CI: 37.7–100.3, *P*<0.001 log-rank test). The degree of malignant epithelial tissue expression of the markers did not correlate with patient survival in the overall cohort, and co-expression did not improve their prognostic value (data not shown). However, further subgroup univariate analysis of stage III patients indicated a significant association between higher CXCL1 expression in the tumour samples and poorer RFS (*P*=0.041). The Kaplan–Meier curve illustrates the poorer outcome of patients with strong CXCL1 immunostaining, relative to moderate or weak/absent expression (Supplementary Figure S2).

Multivariate analysis using Cox regression proportional hazard analysis confirmed disease stage and tumour infiltrate CXCL8 expression as independent predictors of RFS across the study cohort (Table 4). CXCL8-positivity in the tumour infiltrate was associated with a statistically significant reduction in the risk of disease recurrence (hazard ratio: 0.55, CI: 0.36–0.85, *P*<0.001).

## Discussion

In this study, we have conducted an extensive analysis of CXC-chemokine and its receptor expression in CRC tissue using a tissue microarray comprising 254 stage II and stage III biopsies. Intermediate to strong expression of CXCL1, CXCR1 and CXCR2 was concurrently detected in the epithelium of stage II and stage III CRC tumours. In contrast, patient-matched normal epithelial tissue demonstrated predominantly weaker expression of these markers. Although CXCL1 expression has previously been detected in CRC epithelium (Cuenca *et al*, 1992; Baier *et al*, 2005; Wen *et al*, 2006), our analysis showed an additional correlation between CXCL1 and the expression of its biological target, the CXCR2 receptor within the tumour samples (*P*<0.001). Therefore, our study is the first to indicate concurrent ligand and receptor expression in CRC tissue, suggesting a continuous autocrine CXC-chemokine signalling stimulus within these cells.

It is noteworthy that no significant CXCL8 immunoreactivity was detected in the CRC tissue, despite using similar approaches that were previously successful in assessing CXCL8 expression in prostate cancer epithelium (Murphy *et al*, 2005). Previous studies that have characterized CXCL8 expression in CRC tissue have often been conducted in a more advanced disease stage and used techniques other than immunohistochemistry (Ueda *et al*, 1994; Chung and Chang, 2003; Csiszar *et al*, 2004; Erreni *et al*, 2009), however, almost two-thirds of our cohort comprised stage II CRC. Therefore, it is conceivable that the good prognosis of this patient selection may explain the absence of CXCL8 immunoreactivity in the epithelial cells. Instead, we postulate that the expression of CXCL8 may become detectable and especially significant in a more advanced disease stage, indicative of the tumour-promoting effects of the chemokine. Irrespective, the characterisation of CXCL1 and CXCR2 expression in the tumour epithelium provides a functional compensation for the absence of CXCL8. Alternative and more quantitative methods of detection of CXCL8, such assessment of gene expression by PCR, may be of added utility to further investigate these and other chemokines and receptors.

Although extensive analysis was performed, we found no association between CXCR1 and CXCR2 expression and patient outcome in the overall cohort (stage II and stage III tumours). However, a univariate analysis did identify a significant association between elevated CXCL1 expression and poorer relapse-free survival in stage III CRC patients. (*P*=0.04). Although not addressed in this study, de-regulated CXCL1 and CXCL8 signalling has been associated with increased cell proliferation and migratory responses in CRC cells (Brew *et al*, 2000; Itoh *et al*, 2005). Increased secretion of CXCL1 and CXCL8 from tumour epithelium has also been reported to induce angiogenesis in gastro-intestinal cancers (Heidemann *et al*, 2003; Wang *et al*, 2006; Matsuo *et al*, 2009). Furthermore, in Hodgkins’ disease, CXCL1 chemoattraction of inflammatory cells contributes to a complex tumour–host cellular interplay that can result in suppression of cell-mediated cellular immune response and consequently, tumour progression (Skinnider and Mak, 2002). Therefore, it is conceivable that, these individual or combined functions may contribute to the association of CXCL1 expression with an unfavourable outcome in stage III CRC. Understanding the impact of these potential roles of CXCL1 to adverse prognosis merits further studies in CRC collections that include a greater overall number of stage III and/or stage IV tumours.

The previous observation of stage dependence in relation to the prognostic potential of a specific CXC-chemokine's expression raises several important considerations. The impact of CXC-chemokine signalling on clinical outcome is likely to be influenced by numerous factors, including the genetic background of the tumour. For example, increased CXCR2 signalling reportedly underpins oncogenic *Ras*-induced senescence in p53 wild-type tumour cells (Acosta *et al*, 2008), but the effects of oncogenic *Ras*-induced CXC-chemokine signalling may have different effects, for example, in cells harbouring abnormal p53 function. Furthermore, we have shown that stress-induced expression of CXC-chemokines and the magnitude of their signalling effects are more significant in amplitude and duration in PTEN-deficient prostate cancer cells compared with the PTEN-wild-type counterparts (Maxwell and Waugh, unpublished observations). Although unconfirmed, it is plausible that our patient cohort with stage III disease may have different underlying genetic defects than stage II patients; indeed, this concept is currently under investigation through a number of clinical studies (Tejpar *et al*, 2010). Under these circumstances, the increase in CXCL1/CXCR2 signalling may underpin a more adverse clinical impact. More studies in larger cohorts, together with a comprehensive characterisation of the genetic constitution within the tumours would be useful to further explore the merits of our observation.

As alluded to, CXC-chemokine expression within the surrounding tumour microenvironment, distinct from the cancer cell epithelial expression, has been an additional area of investigation. Perhaps the most intriguing observation in this study is the identified impact of peritumoural inflammatory cell CXCL8 expression upon patient outcome. Indeed, positive CXCL8 inflammatory infiltrate expression resulted in enhanced relapse-free survival relative to negative expression, with a hazard ratio of 0.55. This is a considerable improvement in the outcome of a group of patients with an already favourable prognosis (two-thirds stage II disease), and was independent of disease stage on multivariate analysis. Chemokines such as CXCL8 are known to exert potent chemotactic influences on a range of inflammatory cells, such as T cells, neutrophils and basophils (Oppenheim *et al*, 1991), the full implications of which are yet to be fully elucidated and may be relevant to our findings. For example, the prognostic significance of tumour-associated inflammatory cells in gastro-oesophageal cancer is conflicting, with the role of infiltrating macrophages and lymphocytes being reported as both favourable and adverse in contradictory reports (Ma *et al*, 1999; Ohno *et al*, 2003; Koide *et al*, 2004; Deans *et al*, 2006). However, in general the presence of increased tumour-related cellular infiltrate has been associated with improved survival in CRC (Jass, 1986; Nielsen *et al*, 1999; Canna *et al*, 2005); nevertheless, there has been limited characterisation of the expression of pro-inflammatory mediators, including CXC-chemokines within these cells. Our study addressed this question in relation to CXCL8 expression, and provides novel clinical evidence linking such expression specifically within the tumour infiltrate and within these well-defined disease stages to improved patient outcome.

The good prognostic effect conferred by the infiltrate's CXCL8 positivity most likely reflects the immune functions of this chemokine and the anti-tumour role of the inflammatory cells. Although CXCL8 is recognised for its ability to recruit immune cells, the chemokine also induces neutrophil activation promoting the respiratory burst characteristic of these cells. This triggers the release of mediators into the tumour microenvironment, which can facilitate antitumour cytotoxicity (Schroder *et al*, 1990b). Thus, the CXCL8 immunoreactivity detected within the tumour infiltrate may correlate with increased activity of the immune cells. This scenario has been predicted from an experimental model of ovarian cancer in which elevated intra-tumoural CXCL8 expression correlated with an increased infiltration of neutrophils and macrophages and a decreased tumourigenicity (Lee *et al*, 2000). This fascinating observation, therefore, justifies the execution of larger cohort studies to validate our findings. Characterisation of the underlying mechanisms that could explain the observed survival benefit of CXCL8 in this context will also be necessary.

In conclusion, this study demonstrates CXC-chemokine and its receptor expression within CRC tissue. Expression of CXCL1 is associated with a poor prognosis in stage III CRC. Conversely, increased expression of CXCL8 in the inflammatory cell infiltrate of the tumour tissue is associated with a favourable prognosis in this cohort of stage II and stage III CRC tissues. Thus, we demonstrate the potentially distinct effects of chemokine expression and signalling on tumour progression and clinical prognosis within different compartments of the tumour microenvironment.

## Figures and Tables

**Table 1:** Clinicopathologic details of patient cohort

	**No. of patients (*n*=254)**	**%**
*Age (years)*		
Median	64	
Range	35–80	
		
*Gender*		
Male	149	58.7
Female	105	41.3
		
*Tumour site*		
Colon	183	72.0
Rectum	71	28.0
		
*UICC stage*		
Stage II	163	64.2
Stage III	91	35.6
		
*Histological grade*		
I	19	6.3
II	197	77.6
III	30	11.8
Not stated	9	3.5
		
*Received adjuvant treatment*		
Yes	127	50.0
No	127	50.0
		
*Recurrence*		
Yes	163	64.2
No	91	34.8
		
*Status at last review*		
Alive	148	58.2
Dead-all causes	106	41.8
Dead-from CRC	85	33.5

**Table 2:** Relationship between clinico-pathological factors and expression of CXC-chemokine biomarkers in the epithelial cells or immune-infiltrate of colorectal tissue

	**CXCR1 expression (*n*=210)**				**CXCR2 expression (*n*=189)**				**CXCL1 expression (*n*=213)**				**Inflammatory cell CXCL8 expression (*n*=228)**		
	**Weak (*n*=28)**	**Moderate (*n*=160)**	**Strong (*n*=22)**	***P*-value**	**Weak (*n*=18)**	**Moderate (*n*=120)**	**Strong (*n*=51)**	***P*-value**	**Weak (*n*=30)**	**Moderate (*n*=162)**	**Strong (*n*=21)**	***P*-value**	**Negative (*n*=79)**	**Positive (*n*=149)**	***P*-value**
*Age*															
<64	14 (6.7%)	78 (37.1%)	10 (4.8%)		8 (4.2%)	55 (29.1%)	28 (14.8%)		17 (8.0%)	74 (34.7%)	13 (6.1%)		39 (17.1%)	72 (31.6%)	
>64	14 (6.7%)	82 (39.0%)	12 (5.7%)	*0.946*	10 (5.3%)	65 (34.4%)	23 (12.2%)	*0.525*	13 (6.1%)	88 (41.3%)	8 (3.8%)	*0.244*	40 (17.5%)	77 (33.8%)	*0.890*
															
*Gender*															
Male	20 (9.5%)	92 (43.8%)	13 (6.2%)		12 (6.3%)	71 (37.6%)	30 (15.9%)		17 (8.0%)	95 (44.6%)	12 (5.6%)		43 (18.9%)	92 (40.4%)	
Female	8 (3.8%)	68 (32.4%)	9 (4.3%)	*0.383*	6 (3.2%)	49 (25.9%)	21 (11.1%)	*0.821*	13 (6.1%)	67 (31.5%)	9 (4.2%)	*0.975*	36 (15.8%)	57 (25.0%)	*0.322*
															
*Stage*															
II	16 (7.6%)	103 (49.0%)	16 (7.6%)		11 (5.8%)	72 (38.1%)	39 (20.6%)		18 (8.5%)	105 (49.3%)	14 (6.6%)		43 (18.9%)	103 (45.2%)	
III	12 (5.7%)	57 (27.1%)	6 (2.9%)	*0.512*	7 (3.7%)	48 (25.4%)	12 (6.3%)	*0.114*	12 (5.6%)	57 (26.8%)	7 (3.3%)	*0.856*	36 (15.8%)	46 (20.2%)	*0.031*
															
*Site*															
Rectum	5 (2.4%)	43 (20.5%)	11 (5.2%)		6 (3.2%)	28 (14.8%)	12 (6.3%)		8 (3.8%)	48 (22.5%)	2 (0.9%)		16 (7.0%)	47 (20.6%)	
Colon	23 (11.0%)	117 (55.7%)	11 (5.2%)	*0.033*	12 (6.3%)	92 (48.7%)	39 (20.6%)	*0.646*	22 (10.3%)	114 (53.5%)	19 (8.9%)	*0.150*	63 (27.6%)	102 (44.7%)	*0.087*
															
*Received chemotherapy*															
No	15 (7.1%)	75 (35.7%)	13 (6.2%)		8 (4.2%)	53 (28.0%)	24 (12.7%)		15 (7.0%)	81 (38.0%)	11 (5.2%)		46 (20.2%)	71 (31.1%)	
Yes	13 (6.2%)	85 (40.5%)	9 (4.3%)	*0.492*	10 (5.3%)	67 (35.4%)	27 (14.3%)	*0.940*	15 (7.0%)	81 (38.0%)	10 (4.7%)	*0.979*	33 (14.5%)	78 (34.2%)	*0.164*

Values shown in italic are not significant.

**Table 3:** Univariate survival analysis for epithelial CXC-chemokine expression

	**All patients (*n*=254)**				**Stage II patients (*n*=163)**				**Stage III patients (*n*=91)**			
	**Recurrence-free survival**		**Overall survival**		**Recurrence-free survival**		**Overall survival**		**Recurrence-free survival**		**Overall survival**	
	**Recurrences/ no. of patients**	**log-rank (*P*)**	**Deaths/ no. of patients**	**log-rank (*P*)**	**Recurrences/ no. of patients**	**log-rank (*P*)**	**Deaths/ no. of patients**	**log-rank (*P*)**	**Recurrences/ no. of patients**	**log-rank (*P*)**	**Deaths/ no. of patients**	**log-rank (*P*)**
*Tumour stage*												
II	40/163		51/163									
III	51/91	*<0.001*	55/91	*<0.001*								
												
*Tumour epithelial CXCR1*												
Absent/weak	7/28		9/28		6/19		7/19		1/9		2/9	
Moderate	63/160	*0.379*	69/160	*0.586*	59/103	*0.156*	65/103	0.146	4/57	0.675	4/57	0.106
Strong	10/22		11/22		10/14		11/14		0/8		0/8	
												
*Tumour epithelial CXCR2*												
Absent/weak	8/18		9/18		8/13		9/13		0/5		0/5	
Moderate	45/120	*0.748*	47/120	*0.482*	40/74	*0.879*	42/74	0.880	5/46	0.314	5/46	0.698
Strong	18/51		25/51		18/36		23/36		0/15		2/15	
												
*Tumour epithelial CXCL1*												
Absent/weak	9/30		9/30		8/14		9/14		1/16		0/16	
Moderate	63/162	*0.546*	71/162	*0.409*	61/107	*0.585*	66/107	0.628	2/55	0.041	5/55	0.061
Strong	8/21		9/21		6/14		7/14		2/7		2/7	
												
*Tumour inflammatory infiltrate CXCL8*												
Negative	42/79		39/79		38/50		36/50		4/29		3/29	
Positive	42/149	*<0.001*	58/149	*0.150*	41/99	<0.001	54/99	0.029	1/50	0.040	4/50	0.806

Values shown in italic are not significant.

**Table 4:** Multivariate survival analysis for epithelial- and immune infiltrate-localized CXC-chemokine expression

	**Recurrence-free survival**		**Overall survival**	
	**Hazard ratio**	**95% CI (*P*)**	**Hazard ratio**	**95% CI (*P*)**
*UICC stage*				
Stage II	1	1.92–4.62	1	1.60–3.92
Stage III	2.98	(*P*<0.001)	2.50	(*P*<0.001)
				
*Inflammatory infiltrate CXCL8*				
Negative	1	0.36–0.85	1	0.462–1.147
Positive	0.55	(*P*=0.007)	0.728	(*P*=0.171)
