# Supplementary material for: The expression and prognostic impact of CXC-chemokines in stage II and III colorectal cancer epithelial and stromal tissue
Source: Br J Cancer. 2011 Feb 1;104(3):480–7. doi: 10.1038/sj.bjc.6606055 (PMC3049559; doi:10.1038/sj.bjc.6606055)
Supplement: Supplementary Figures & Tables S1 and S2 [file 6606055x1.ppt]

## Slide 1
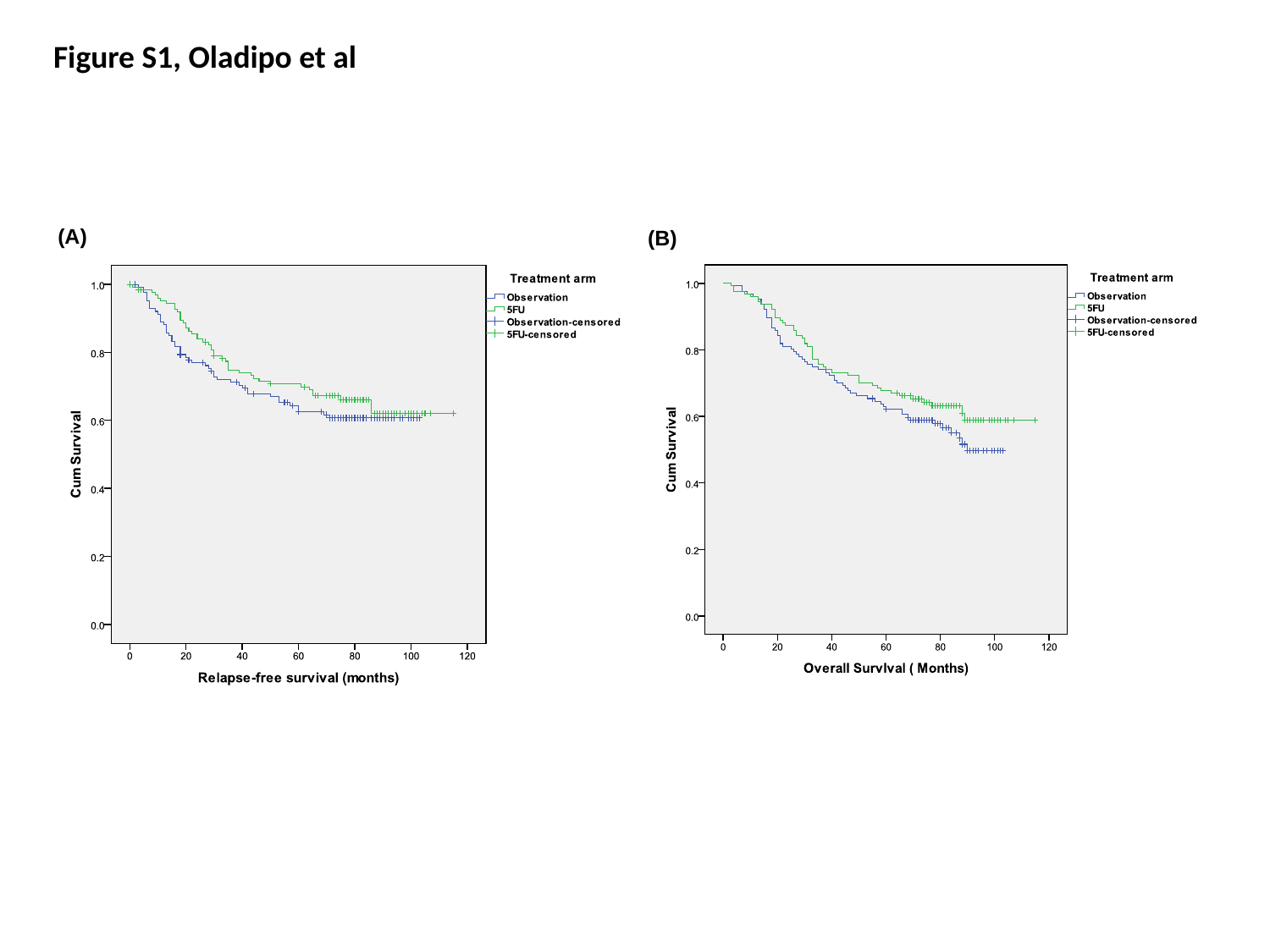

Figure S1, Oladipo et al
(A)
(B)

## Slide 2
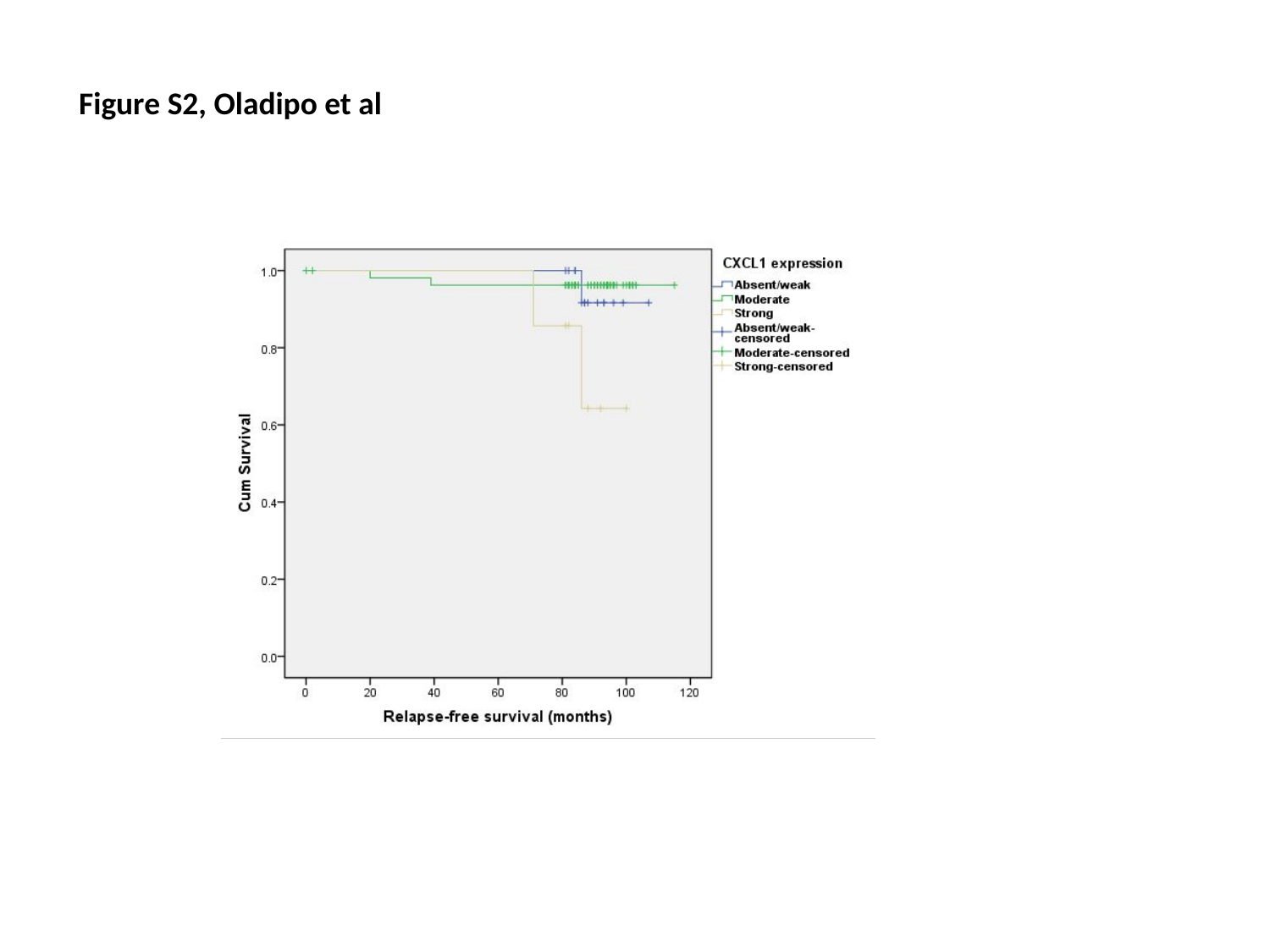

Figure S2, Oladipo et al

## Slide 3
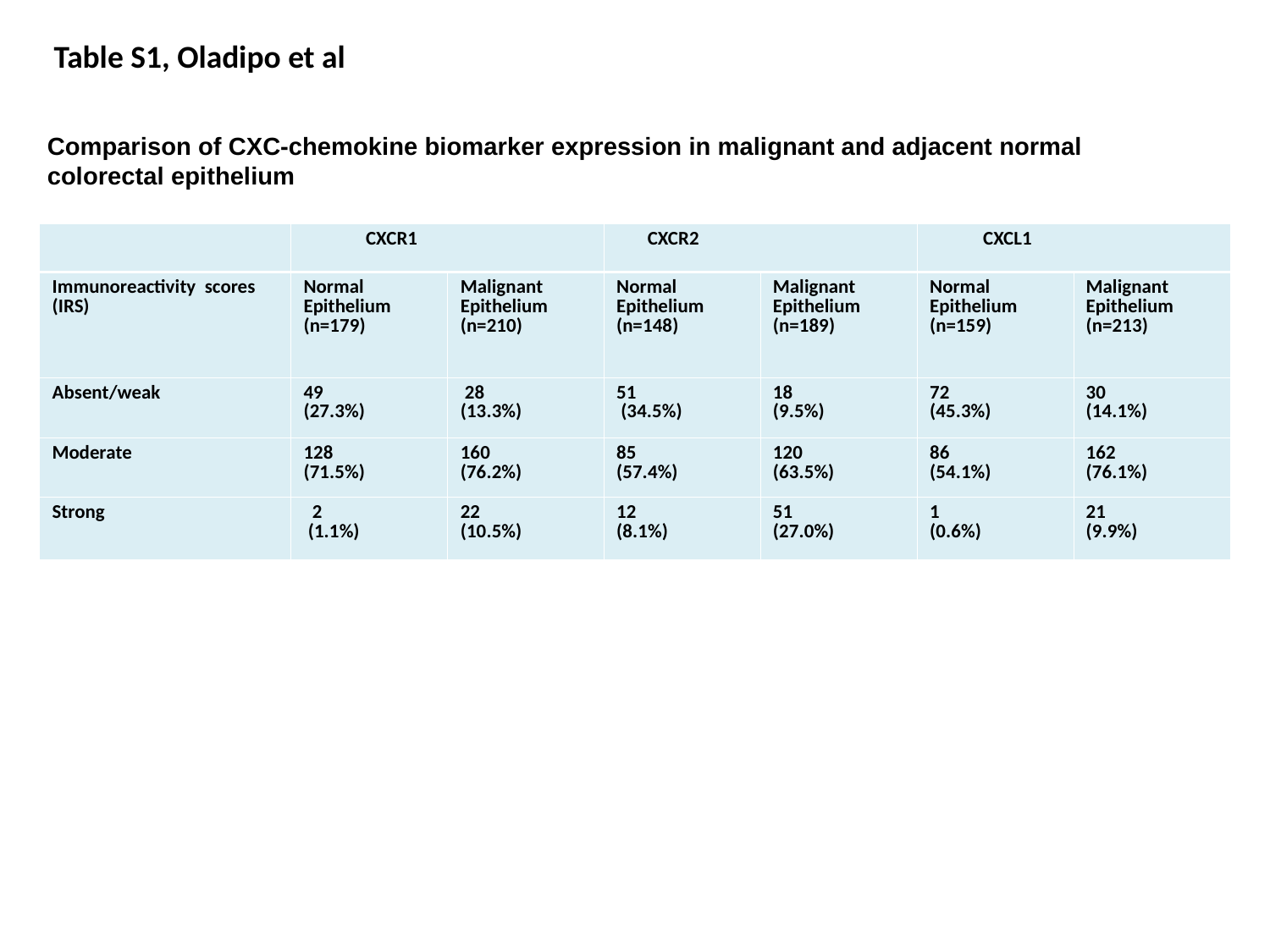

Table S1, Oladipo et al
Comparison of CXC-chemokine biomarker expression in malignant and adjacent normal colorectal epithelium
| | CXCR1 | | CXCR2 | | CXCL1 | |
| --- | --- | --- | --- | --- | --- | --- |
| Immunoreactivity scores (IRS) | Normal Epithelium (n=179) | Malignant Epithelium (n=210) | Normal Epithelium (n=148) | Malignant Epithelium (n=189) | Normal Epithelium (n=159) | Malignant Epithelium (n=213) |
| Absent/weak | 49 (27.3%) | 28 (13.3%) | 51 (34.5%) | 18 (9.5%) | 72 (45.3%) | 30 (14.1%) |
| Moderate | 128 (71.5%) | 160 (76.2%) | 85 (57.4%) | 120 (63.5%) | 86 (54.1%) | 162 (76.1%) |
| Strong | 2 (1.1%) | 22 (10.5%) | 12 (8.1%) | 51 (27.0%) | 1 (0.6%) | 21 (9.9%) |

## Slide 4
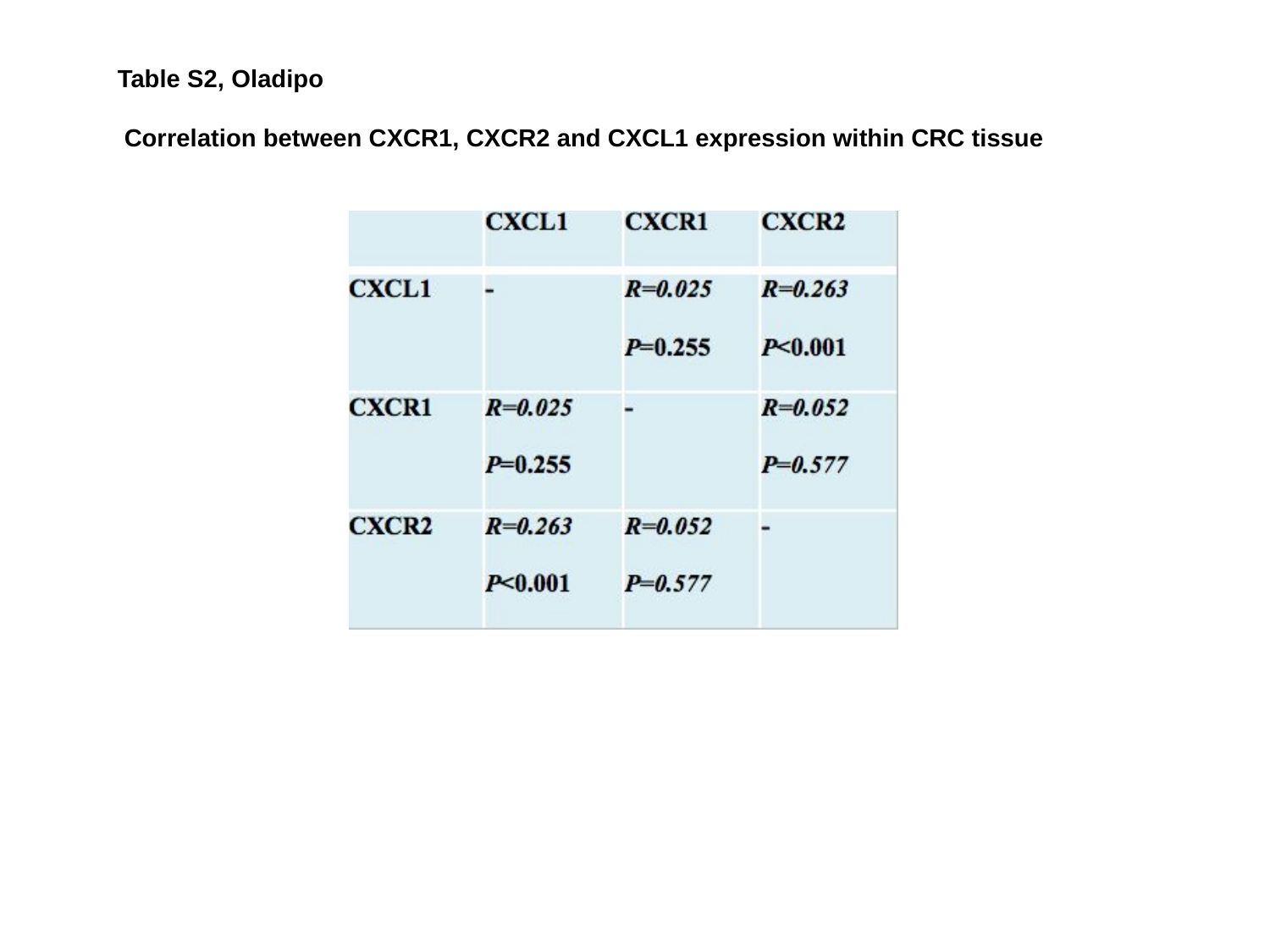

Table S2, Oladipo
 Correlation between CXCR1, CXCR2 and CXCL1 expression within CRC tissue
