# Supplementary material for: The expression and prognostic impact of CXC-chemokines in stage II and III colorectal cancer epithelial and stromal tissue
Source: Br J Cancer. 2011 Feb 1;104(3):480–7. doi: 10.1038/sj.bjc.6606055 (PMC3049559; doi:10.1038/sj.bjc.6606055)
Supplement: Supplementary Figure Legends [file 6606055x2.doc]

**Supplementary Figure S1. Relapse-free and overall survival of stage II and III CRC patients according to treatment arm**. (A) Kaplan Meier curve representing the recurrence-free survival of stage II and stage III colorectal cancer patients stratified on the basis of post-operative treatment received in the adjuvant study. Median survival was not reached by either arm of the trial, and there was no difference in the survival curves of patients who received adjuvant 5FU or observation only (P=0.227, log ranks test). (B) Kaplan Meier curve also indicating no difference in the overall survival in each arm of the study (P=0.381). Again median survival was not reached in either arm.

**Supplementary Figure S2. Relapse-free survival of stage III CRC patients according to CXCL1 expression** Kaplan Meier curve illustrating the recurrence-free survival of stage III CRC patients stratified according to the mean CXCL1 immunoreactivity score. Patients with strong CXCL1 demonstrate a poorer recurrence-free survival compared with moderate or weak/absent expression (P=0.041, log ranks test).
